# Intussusception reveals MUTYH-associated polyposis syndrome and colorectal cancer: a case report

**DOI:** 10.1186/s12885-019-5505-8

**Published:** 2019-04-05

**Authors:** Gustavo Heluani Antunes de Mesquita, Bárbara Justo Carvalho, Kayo Augusto de Almeida Medeiros, Fernanda Nii, Diego Ramos Martines, Leonardo Zumerkorn Pipek, Yuri Justi Jardim, Daniel Reis Waisberg, Marcos Takeo Obara, Roberta Sitnik, Alberto Meyer, Cristóvão Luis Pitangueiras Mangueira

**Affiliations:** 1 FMUSP, São Paulo, Brazil; 2 Departamento de Gastroenterologia, Hospital das Clínicas, HCFMUSP, São Paulo, Brazil; 3 Hospital Israelita Albert Einstein, São Paulo, Brazil; 4 Diretor do Departamento de Patologia Clínica e Anatomia Patológica do Hospital Israelita Albert Einstein, São Paulo, Brazil

**Keywords:** MUTYH-associated polyposis, Intussusception, Hereditary colorectal cancer

## Abstract

**Background:**

We are reporting a rare case of MUTYH-associated polyposis, a colorectal cancer hereditary syndrome, diagnosticated after an intussusception. Colorectal cancer is an important cause of cancer related mortality that can be manifested by an intussusception, a rare occurrence in adults and almost always related to tumors. Approximately 5% of colorectal cancers can be attributed to syndromes known to cause hereditary colorectal cancer, such as MUTYH-associated polyposis, autosomal genetic syndrome associated with this disease.

**Case presentation:**

We present the case of a 44 years old male, that sought medical consultation with a complaint of abdominal discomfort, that after five days changed its characteristics. The patient was sent to the emergency department were a CT-scan revealed intestinal sub-occlusion by ileocolic invagination. Right colectomy was carried out. The anatomic-pathological examination revealed a moderately differentiated mucinous adenocarcinoma and multiples sessile polyps, which led to the suspicion of a genetic syndrome. In the genetics analysis two mutations were observed in the MUTYH gene, and MUTYH-associated polyposis was diagnosticated.

**Conclusion:**

This case demonstrates the importance of meticulous analysis of the patient examinations results to identify possible discrete alterations that can lead to improved understanding of disease.

## Background

Colorectal cancer is the fourth most common, and the second biggest cause of cancer related mortality in the United States [1], with an estimated 1.4 million new cases diagnosed worldwide in 2012 [2]. Colorectal cancer predominantly affects males, and its incidence increases with age. Despite this, Bailey et al [3] highlight an increasing incidence in younger patients, between 20 and 34 years of age, which could increase by 90% for colon cancer and 124.2% for rectal cancer by 2030. Obesity, diets rich in red meat and processed foods, tobacco consumption, alcoholism and the presence of inflammatory intestinal diseases are considered risk factors for incidence of colorectal cancer [4].

Approximately 30% of colorectal cancers present a relationship with inherited genetic factors, and 5% are attributed to syndromes known to cause hereditary colorectal cancer [5]. Among these, we can highlight syndromes caused by mutations in the APC gene, such as familial adenomatous polyposis and Gardner syndrome; mutations in the DNA repair system, such as Lynch syndrome, mutations in the MUTYH gene, such as MUTYH-associated polyposis (MAP); as well as more uncommon syndromes such as type X colorectal cancer and Peutz-Jeghers syndrome [6].

MAP is an autosomal genetic syndrome associated with colorectal cancer. Carriers of the syndrome have a 43 to 100% lifetime risk of developing colorectal cancer. Diagnosis of the syndrome is made through discovery of biallelic pathogenic mutations of the MUTYH gene [7, 8].

Clinical presentation of the syndrome is highly variable; patients can present no polyps, up to hundreds of polyps with variable morphology [9, 10]. Duodenal and gastric polyps may be present, as well as an increased risk of ovarian, bowel, breast and endometrial cancers. Another possible characteristic is the presence of tumors in the sebaceous glands, skin and other appendages, dental abnormalities and hypertrophy of the retinal pigment epithelium [11].

Patients with colorectal cancer can be asymptomatic, where the disease is detected via screening. They can also present symptoms and suspicious signs, such as blood in stools, weight loss, alteration of intestinal habits, abdominal pain and anemia; or even being admitted to emergency units with intestinal obstruction, peritonitis and acute gastrointestinal bleeding [12]. One of the forms of intestinal obstruction is intussusception, rare in adults, and almost always related to tumors [13, 14].

The objective of this article is to present the case of a patient diagnosed with MAP following intestinal intussusception associated with a colon adenocarcinoma.

## Case presentation

A patient of male sex, 44 years of age, sought medical consultation with complaints of heartburn, epigastric fullness and regurgitation for approximately 15 days, which he related to diet and anxiety. The patient did not report nausea, vomiting or alteration of intestinal habits. He used pantoprazol without improvement. As a comorbidity, the patient presented dyslipidemia. As well as the symptoms complained about, the patient also presented multiple sebaceous cysts. There was no relevant family history.

**Fig. 1:**
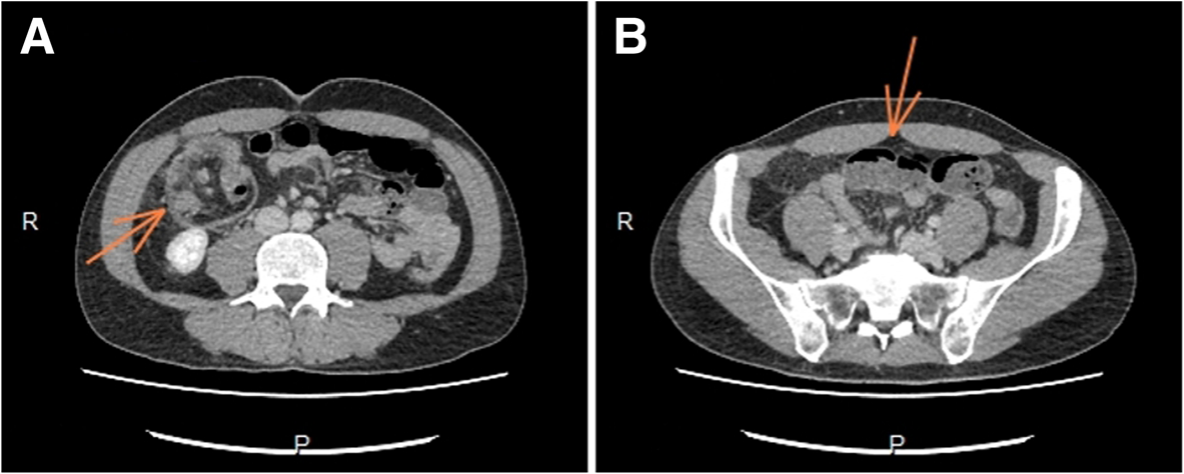
Image from computerized tomography demonstrating **a**- ileocolic intussusception **b** – distention of the small intestine

Dyspeptic syndrome was suspected, and an upper digestive endoscopy and abdominal ultrasonography were requested. After five days, the patient presented alteration of symptoms, and was sent to the emergency department, where he reported colic pain of moderate intensity localized in the right flank and right iliac fossa. Abdominal computerized tomography was carried out, which revealed intestinal sub-occlusion by ileocolic invagination, being extended to the beginning of the transverse colon. Also, the cecal appendix was thickened (Fig. 1).

**Fig. 2:**
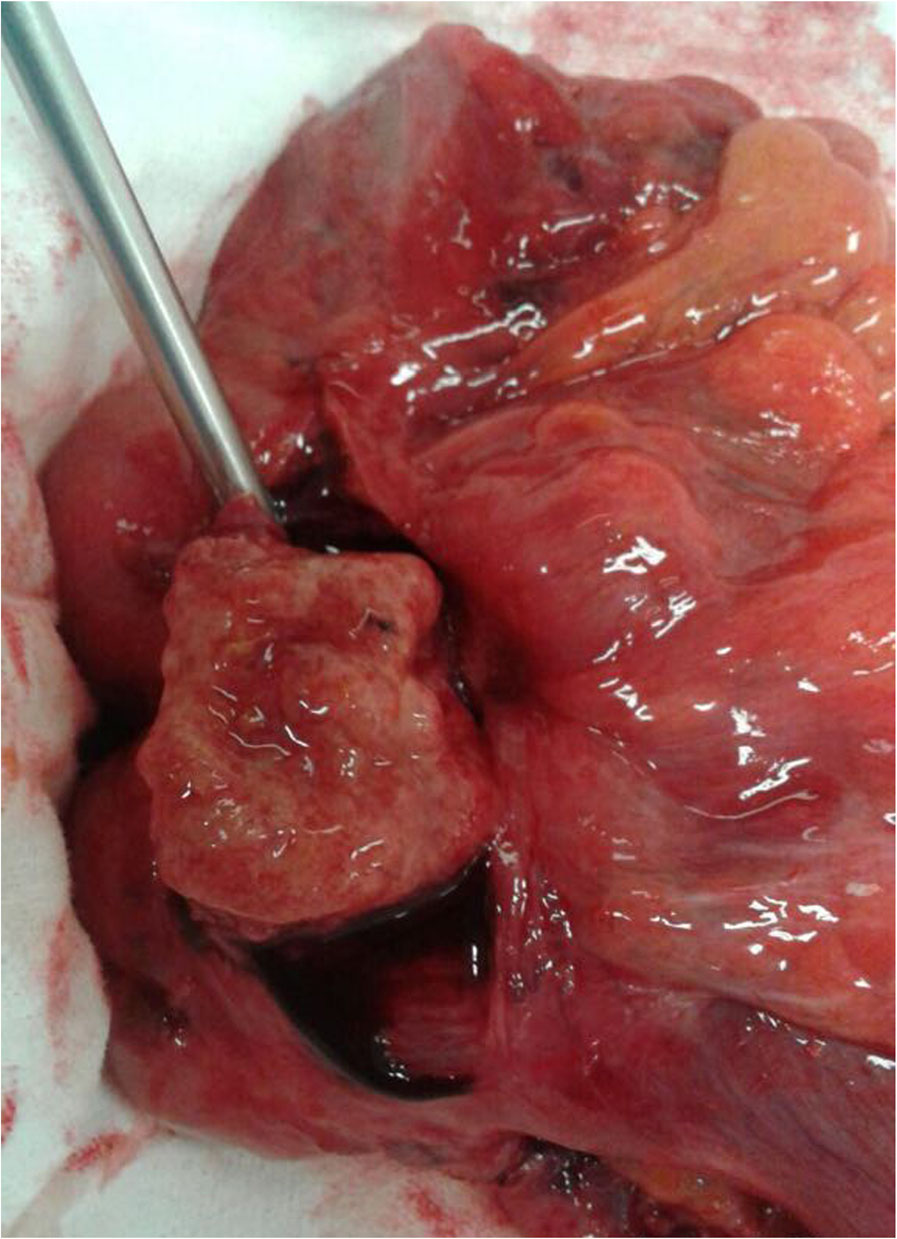
Open cecum showing the tumor in its lumen

Following diagnosis, surgical intervention to undo the invagination through laparoscopy was carried out, followed by right colectomy. The anatomic-pathological examination revealed a moderately differentiated mucinous adenocarcinoma (low grade), T3 N0 (0/39 lymph nodes evaluated) (stage 2A). Multiple sessile polyps measuring between 2 mm and 6 mm were also found, which led to the suspicion of a genetic syndrome associated with colorectal cancer (Fig. 2).

An immunohistochemical analysis of the tumor cells initially revealed immunoexpression of preserved proteins in the repair genes (MLH-1, MSH-2, MSH-6, PMS2), ruling out association with a microsatellite instability phenotype. A genetic panel was then used for genes associated with intestinal polypoid cancer. The APC and MUTYH genes were analyzed using a library prepared with a multiplexed PCR kit (Qiagen) and a library using conventional PCR and Nextera (Illumina). Both libraries were submitted to Next Generation Sequencing (NGS) on a MiSeq Sequencer (Illumina). Paired-end reads were aligned to a UCSC reference sequence (hg19) and processed with a bioinformatics pipeline developed in the laboratory. Both genes were completely sequenced, with at least 50X coverage in 100% bases, including all exons and 10 bp of adjacent intronic regions.

Genetic analysis revealed an absence of mutations on the APC gene, excluding syndromes like familial adenomatous polyposis (FAP), attenuated FAP and Gardner syndrome [15]. There were two variants found on the MUTYH gene. The alterations found were c.536A > G p.(Tyr179Cys) on exon 7 and c.1147delC p.(Ala385Profe*23) on exon 12, both of them classified as pathogenic on ClinVar database. This genetic constitution (Table 1) is related to MUTYH-associated polyposis (MAP).

**Table 1:** Details of variants found in MUTYH gene

Gene	Variant	Exon	Population frequencie	Zigosity	Classification
MUTYH (NM_001128425)	c.536A > G p.(Tyr179Cys)	7	0,2153%	Heterozygous	Pathogenic variant
MUTYH (NM_001128425)	c.1147delC p.(Ala385Profs*23)	12	0,00752%	Heterozygous	Pathogenic variant

A diagnosis of a genetic syndrome related to colorectal cancer was made, and follow up according to the American Cancer Society guidelines was indicated. The first checkup was made three months after surgery, with thoracic and abdominal tomography, upper digestive endoscopy, colonoscopy and dosage of CEA. The results were within normal bounds, with a CEA of < 0.5 and the presence of 6 tubular adenomas with a low degree of dysplasia in the colonoscopy, which were excised. Other colonoscopies were made 6 months after surgery and 1 year after surgery, resulting in the excision of 9 and 3 tubular adenomas with a low degree of dysplasia, respectively.

## Discussion

MAP is a rare syndrome, responsible for less than 1% of cases of colorectal cancer [9], and despite high lifetime risk of developing colorectal cancer, these patients have a much higher 5-year survival rate than patients with sporadic colorectal cancer [16]. The clinical presentation of patients with MAP is highly heterogeneous, with a variable number and types of polyps. Our 44 year old patient had a form with few polyps, multiple sebaceous cysts, and no family history of the disease.

The diagnosis of colorectal cancer in our patient was made following 15 days of discomfort similar to dyspeptic syndrome. Abdominal pain, rare as the only symptom of colorectal cancer [17], intensified and changed, leading the patient to seek medical attention. A diagnosis of ileocolic intussusception was made, a rare condition in adults, but that in between 54.5 and 93.8% [18–21] of cases is related to tumors.

Only 5% of intussusceptions occur in adults [14], being a rare occurrence. In the literature there is much disagreement about the percentage of cases that can be attributed to tumors, and the proportion of malignant tumors among them. In colonic intussusceptions, the percentage of malignant tumors is as high as 90% [20]. Our patient is an example of intussusception being a manifestation of colorectal cancer. Due to the presence of multiple polyps on the surgical specimens, tests for genetic syndromes related to this malignancy were requested.

Case reports in the literature, such as ours, associate intussusceptions with tumors on the colon. Xie-qun [22] et al. and Gayatri Asokan [23] et al. report cases of patients that present to emergency services with obstructive symptoms, receiving a diagnosis of intussusceptions, and in surgery, tumors were considered of organic cause. Beyond the absence of diagnosed genetic syndromes, such cases differed from ours because of the patients’ profiles; an older person in the first case and a patient with a more pronounced clinical presentation in the second, with rectal bleeding. Two other cases are reported by Manish Chand [24] et al., although the patients received diagnosis of colorectal cancer prior to that of intussusception, which occurred only in the intraoperative phase. In none of these cases was there suspicion of a genetic syndrome.

Schirier JC [25] et al. report MAP diagnosed in a 14 year old patient following intussusceptions. It is notable that in this age group, intussusception is a more frequent clinical manifestation [26], possibly due to the higher predisposition for intussusception in infancy. Such patients should be tested for genetic syndromes, and differ from our case due to the young age at which the patients develop tumors.

Jenifer M. Dan [27] et al. and Ryo Inada et al. [28], both present cases of intussusceptions in which the presence of genetic syndromes is associated with colorectal cancer. Both cases were young patients; 27 years old and 24 years old respectively. In the first case, due to the absence of synchronic polyps, the hypothesis was disregarded and genetic testing was not carried out. In the second case, analysis was carried out only for Lynch syndrome. In our patient, suspicion of genetic syndrome was raised not by the age of the patient, but because of the presence of synchronic polyps in the surgical specimen, from which a diagnosis of MAP was made. In related studies, genetic syndromes were possibly not diagnosed because the tests were not completely carried out. In the first case, the patient could have presented an attenuated form, without the presence of synchronic polyps, while in the second, other syndromes not tested for could have been diagnosed. A case reports summary is available in Table 2.

**Table 2:** literature case reports summary

Case Report	Clinical presentation	Genetic Syndrome and Mutations
Xie-qun et al	76yo male patient presented to emergency service with obstructive simptoms	None
Gayatri Asokan et al	36yo female patient presented to emergency service with obstructive simptoms	None
Manish Chand et al	Adult patients diagnosed with colorectal cancer, with intraoperative intussusception	None
Schirier JC et al	14yo male patient presented to emergency service with obstructive simptoms	Mutyh associated polyposis - mutations not specified
Jenifer M Dan et al	27yo male patient with intermitent intussuception	None
Ryo Inada et al	24yo male patient with abdominal pain and tenesmus	Tested negative for Lynch Syndrome

Patients with hereditary syndromes related to colorectal cancer needs accurate colonoscopy examinations because of the risk of polyps developing into colorectal cancer. Therefore, the completion rate of a colonoscopy is an important aspect, since that it guarantees a full examination of patients colon, increasing the chance of all polyps excision [29, 30]. In our case report, the patient was submitted to colonoscopy 3 months, 6 months, and 1 year after surgery, with the excision of 6, 9 and 3 tubular adenomas with a low degree of dysplasia, respectively.

Our case presented some particularities that differed from other reported cases, as it was an adult patient diagnosed with a genetic syndrome and colorectal cancer following intussusception. It is notable that the patient presented an attenuated form of polyposis and the presence of multiple sebaceous cysts, one of the clinical variants of MAP. An important aspect is that the diagnosis was only possible because there was a meticulous analysis of the patients anatomic-pathological exam, where multiple sessile polyps were found. This finding led to the suspicious of an hereditary syndrome. The perseverance in the genetic analysis after negative results for FAP, attenuated FAP and Gardner syndrome is another important aspect, since MAP was only diagnosed because it was decided to test for other rare syndromes related to colorectal cancer.

## Conclusion

This case demonstrates the importance of meticulous analysis of the patient examinations results to identify possible discrete alterations that can lead to improved understanding of disease, and tailored treatment for patients accordingly. The discovery and analysis of polyps led to the diagnosis of an underlying genetic condition that altered follow up strategy for the patient.

## Abbreviations

CEA: Carcinoembryonic antigen
FAP: Familial adenomatous polyposis
MAP: MUTYH-associated polyposis
